# Editorial: Bioinspired superwettable materials from design, fabrication to application, volume Ⅱ

**DOI:** 10.3389/fbioe.2023.1249653

**Published:** 2023-07-18

**Authors:** Zhihao Shang, Ran Zhao, Feilong Zhang, Jingxin Meng

**Affiliations:** ^1^ Yanshan University, Qinhuangdao, China; ^2^ Technical Institute of Physics and Chemistry, Chinese Academy of Sciences (CAS), Beijing, China; ^3^ University of Chinese Academy of Sciences, Beijing, China; ^4^ Nanyang Technological University, Singapore, Singapore; ^5^ Binzhou Institute of Technology, Weiqiao-UCAS Science and Technology Park, Binzhou, China

**Keywords:** bioinspired, anti-adhesion, adhesion, bone tissue recovery, wound healing, sensing detection

Superwettable materials, which combine the surface micro/nano structure and chemical composition, have already emitted light and heat in many fields, such as anti-fouling, signal monitoring, rehabilitation treatment, etc. Special biological phenomena in nature constantly provide inspiration for the design of superwettable materials, for example, self-cleaning materials inspired by lotus leaves, adhesive materials with superwettability inspired by barnacles, and signal recognition systems inspired by mammalian olfactory recognition. We believe that more biological phenomena can be explored and more applicable superwettable materials can be provided.

The primary task of this Research Topic is to compile high-quality researches on bioinspired superwettable materials for conquering real-life scientific problems. This Research Topic contains nine articles including six research articles and three review articles from world-leading scientists in various fields, aiming to emphasize practical evolution of bioinspired materials with superwetting properties in the fields such as fouling prevention, bone tissue recovery, wound healing and sensing detection.

Regulating the adhesion properties of various superwetting materials is a key foundation for their applications. Barnacles, one of the most common living organisms in the ocean, exhibit high underwater adhesion strength with the substrate due to their hydrogen bonding and electrostatic interaction. Inspired by this, Hao et al. reported a hydrogel adhesive composed of polyethylene imine and polymethacrylic acid, achieving high adhesion strength under water via overcoming the adverse effects of water layer. The adhesion strength under water or other solvents remains at high levels of 1.99 and 2.70 MPa, respectively. As a widely used liquid organic salt, ionic liquid has irreplaceable advantages such as negligible vapor pressure, adjustability and lubricity, etc. To develop bioinspired superwettable materials, Zhang et al. summarized the design of bioinspired ionic-liquid based interfacial materials and then, exemplified their latest applications in the field of adhesion (e.g., wound dressing) and anti-adhesion (e.g., anti-scaling).

Repairing bone injuries has attracted much attention in the medical fields. To accelerate the healing of injured bone tissue, it is urgent to find powerful biocompatibility bone grafting materials. For example, Liu et al. proposed a bioinspired strontium doped magnesium phosphate cement (S-MPC) to solve sufficient biocompatibility which is the biggest shortcoming of MPC. A certain amount of strontium was introduced into the MPC, endowing the S-MPC with the advantages of high crystallinity, excellent biocompatibility and stable mechanical strength. S-MPC is not only beneficial to accelerate bone tissue repair, but also can meet different needs of patients with bone injury by changing its shape. In addition, the absorption of alveolar bone caused by dental surgical procedure is an irreversible bad phenomenon. To solve this problem, Guo et al. prepared the cell microspheres for bone repair by introducing mesenchymal stem cells and vascular endothelial cells into the polyether F127 diacrylate hydrogel. The autonomous diffusion ability of the fabricated cell microspheres can fully contact bone tissue defects in a three-dimensional manner, showing great biocompatibility and excellent mechanical properties.

As well known, skin is the largest protective organ of the human body, but skin replacement would be an inevitable solution if the damage area of the skin is too large. Skin transplantation means immune rejection and other problems, so bioinspired skin has attracted extensive attention in the field of rehabilitation medicine. Wang et al. summarized the classification of bioinspired skin, and then concluded that the characteristics of electronic skin are stimulus response and self-healing ability. More importantly, this review discussed the practical application of bioinspired skin. Therefore, the development of bioinspired superwettable materials has opened new avenues for bioinspired skin. Additionally, wound region caused by skin tissue damage occur from time to time. Controlling the amount of bleeding is identified as the primary task in the four periods of wound healing. Based on this, Zhong et al. summarized the latest progress in superwettable wound dressings and provided a detailed introduction to different materials used for hemostasis and exudate management. Moreover, the management of exudates is also an important means related to wound healing which can avoid the continuous inflammation of the wound. For instance, Lan et al. fabricated an enhanced fractal self-pumping (EFS) hydrogel which can quickly absorb water and discharge it in 0.4 s owing to the introduction of hollow glass microspheres in the hydrogel precursor. By simulating human skin wound models in animal experiments, EFS hydrogel wound dressing can significantly accelerate wound recovery. It is believed that above articles will make readers enthusiastic about learning wound management and are expected to provide design ideas for interface materials for biomedical applications.

In the field of polymer-based organic vapor sensors, higher accuracy and lower detection limit are urgently needed. Inspired by mammalian olfactory recognition system, Zhao et al. proposed a bioinspired polymer array vapor sensor that combines principal component analysis algorithm, which can classify and recognize a variety of common organic compounds (e.g., ethanol, acetone, methylene chloride). The detection ability of the sensor is attributed to the introduction of aggregation-induced emission molecules into the polymer. Malathion is a traditional organophosphorus insecticide component, but the abuse of malathion will lead to life health and ecological environment problems. In order to achieve the detection goal of malathion, Li et al. designed an efficient sensor for malathion with the detection limit of 1.48 g/L and the change can be easily identified by the naked eye. In summary, the above sensors with extremely high accuracy and very low detection limit, are expected to be used for environmental monitoring, agricultural production, medical treatment, and other fields.

Overall, this Research Topic cover several high-profile fields in bio-inspired materials with super wettability, such as fouling prevention, bone tissue recovery, wound healing and sensing detection, which make readers enthusiastic about learning the latest advances in bio-inspired superwettable materials. Although exciting results are achieved one after another, more bioinspired superwettable materials will come into practical applications soon.

